# A Review on Preparation and Applications of Silver-Containing Nanofibers

**DOI:** 10.1186/s11671-016-1286-z

**Published:** 2016-02-09

**Authors:** Shu Zhang, Yongan Tang, Branislav Vlahovic

**Affiliations:** Department of Mathematics and Physics, North Carolina Central University, Durham, NC 27707 USA

**Keywords:** Silver nanoparticle, Biomaterial, Polymer nanofiber, Electrospinning

## Abstract

Silver-containing nanofibers are of great interest recently because of the dual benefits from silver particles and nanofibers. Silver nanoparticles are extensively used for biomedical applications due to the antibacterial and antiviral properties. In addition, silver nanoparticles can excite resonance effect of light trapping when pairing with dielectric materials, such as polymer. Comparing to the traditional fabrics, polymer nanofibers can provide larger number of reaction sites and higher permeability contributed to their high surface-to-volume ratio and high porosity. By embedding the silver nanoparticles into polymer nanofiber matrix, the composite is promising candidates for biomaterials, photovoltaic materials, and catalysts. This work demonstrates and evaluates the methods employed to synthesize silver nanoparticle-containing nanofibers and their potential applications.

## Review

Due to their antibacterial [1–3], fungicidal [4–6], and antiviral [7–10] properties, silver particles have drawn tremendous academic and industrial interests. Besides of the benefits of their biomedical applications, when the size of silver particles decreases to nanoscale, the high surface area and surface energy make silver nanoparticles one of the best candidates for catalysis and optical absorption. The silver nanoparticles enable surface plasmon resonances when being placed in dielectric environment, leading to higher optical absorption efficiency [11]. Therefore, the applications of silver nanoparticles expand from biology, electronics, textile industries, and catalysis material to optical and photovoltaic materials, such as solar cells. Fibers with nanostructures have also been intensively investigated because of its high surface-to-volume ratio. Compared to traditional materials, membranes and films composed of nanofibers have extremely high surface area and nanometer grade micropores, which can provide both larger number of reactive sites for chemicals and excellent filtration property and breathability when being used as insulating fabrics and biomaterials such as wound dressing and protective textiles.

With the merits of both advantages of antibacterial and optical enhancing properties of silver nanoparticles, and high surface area and surface energy of nanofibers, silver-containing nanofibers can be directly used as films, coatings, and fibers in electronics, sensors, multispectral filters, catalytic materials, water treatment, and nanopaints. Although a variety of metal nanoparticles including Au, Pt, and Pd have been prepared in organic solvent with the presence of different polymers, such as polyvinylpropelene (PVP) and polyacrylic acid (PAA), there are handful paper published reporting the preparation process of silver nanoparticle-containing polymer nanocomposites. In response to the rapid growing interest in this type of materials, this work is focusing on critical review of the synthesis and applications of silver particle-containing nanofibers.

### Synthesis of Silver Nanoparticles

Depending on the reducing sources and methods, there are mainly two methods to prepare silver nanoparticles. Physical routes normally employ laser impulse energy to reduce silver from bulk to atoms and ions. Chemical methods utilize reducing agents to reduce silver ions from their precursors into metallic silver atoms.

#### Physical Method

**Fig. 1:**
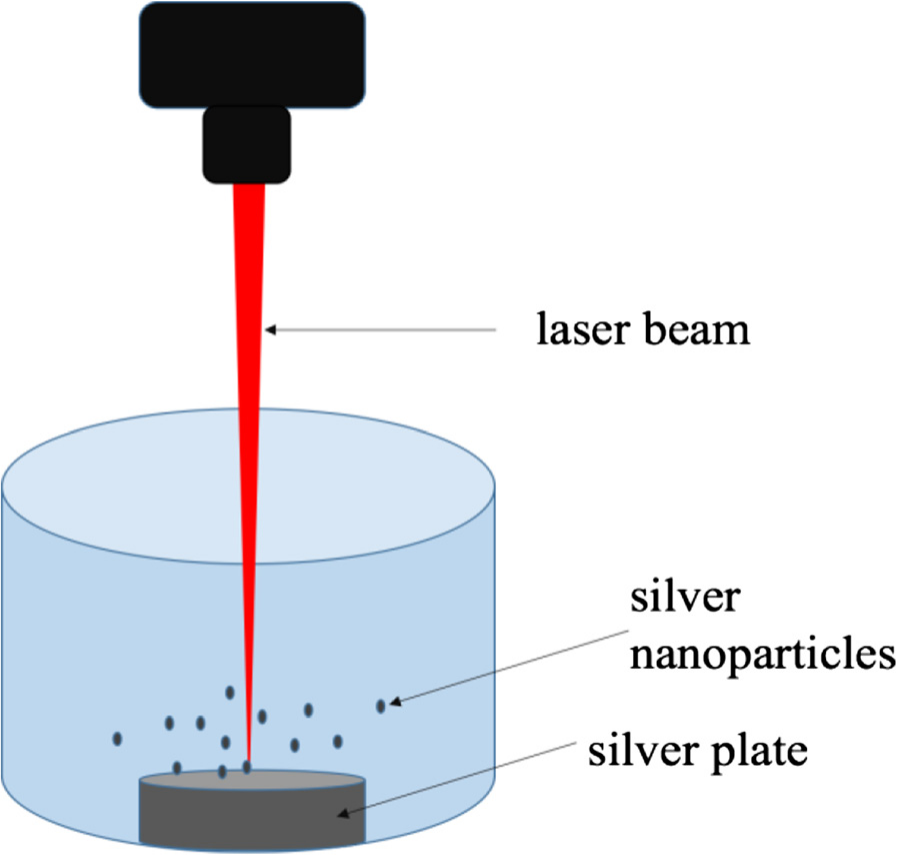
Schematic of experiment setup for silver nanoparticle production with laser ablation

One major route to obtain silver nanoparticles is physical method utilizing dispersion/condensation and laser ablation from metallic bulk materials in liquid solution (Fig. 1). Cotton-Chumanov [12] and Fojtik [13] proposed a method to produce silver nanoparticles from silver plates immersed in liquid phases and illuminated by high-energy laser beam. This method is the most extensively used method nowadays. Researchers, i.e., Mafune [14, 15] and González-Castillo [16] postulate that the metal plate absorbs a great part of the laser impulse energy and forms a hot plasma containing a high concentration of silver atoms and ions. The liquid phase serves as a cooling medium to cool down the heat from plasma. Hence, the physicochemical properties of the solution affect the rate of nanoparticle formation, their shape, size, and polydispersity [17].

One of the advantages of using laser ablation is that no chemical agents are needed in this process; therefore, no additional purification is required for the obtained silver nanoparticle colloids, and it can be directly used for further applications. Although this method has been widely developed and investigated, there are still some contradictories on the relationship between the silver nanoparticle sizes and power of laser beam. Generally, most reported work shows that the size of nanoparticles increases linearly with the power of laser beam [12, 18–20].

Another physical method is evaporation and condensation. It is normally carried out in a tube furnace at room atmospheric pressure [21]. Various metallic particles have been prepared using this technique such as Au, Ag, and PbS [22]. In the evaporation/condensation process, the primer of silver-containing organics is placed in the boat of the heating center. After the other non-silver elements are evaporated and carried away by the carrier gas, leaving only the silver particles in the furnace, the evaporation/condensation technique is easy and straightforward; however, the major drawback of this technique is large energy consumption and slow process.

In addition to the abovementioned methods, another extensively used method is electrochemical route, where silver nanoparticles are prepared in a special electrochemical cell [23]. In this electrochemical cell, an external electric field is applied to silver anode. The silver ions are reduced on platinum cathode and form clusters, which subsequently become silver nanoparticles. This method can be conducted at room temperature, and the size of silver nanoparticles can be controlled by the current density [24].

#### Chemical Method

Chemical reduction is the most employed way to prepare silver nanoparticle as stable colloidal dispersion in water and other organic solvents. Many studies have shown that after the silver ions are reduced to silver atoms, they will further agglomerate into oligomeric clusters, leading into the formation of silver nanoparticles. Normally, to produce silver nanoparticles, the most frequently used precursors for chemical reduction are silver nitrate [25–28], silver acetate [29, 30], silver citrate [30–32], and silver chlorate [30, 31, 33]. Among various reducing agents, the most commonly used reductants are borohydride, citrate, ascorbate, and compounds with hydroxyl or carboxyl groups such as alcohol, aldehydes, carbohydrates, and their derivatives [34–37].

It has been found that the growth mechanism of silver nanoparticles is strong influenced by the properties of reducing agents. Strong reducer composition, such as borohydrates, generates monodispersed silver nanoparticles with larger sizes, while the mild reductants such as ascorbate and citrate create smaller numbers of nuclei at a slow rate, which in turns promotes the generation of silver nanoparticles with smaller sizes and wider dispersion [38, 39].

It is also interesting to mention that the dispersion medium plays an important role in particle sizes and morphologies of silver nanoparticles during chemical reduction. The dispersion medium, which refers to the solution and solvent system, serves as a protective agent, which can be absorbed or bind onto the particle surface, to avoid the agglomeration of the particles [40, 41]. Polymers are best candidates for stabilizing the silver nanoparticles. Poly(vinylpyrolidone) (PVP), poly(methylacrylic acid) (PMAA), poly(methylmethacrylate) (PMMA), and poly(ethylene glycol) (PEG) are the mostly used polymers as stabilizer.

#### Other Methods

Besides the physical and chemical methods, other alternative techniques have also been employed to synthesize silver nanoparticles including biological and photochemical routes. In the case of biological synthesis, natural sources such as plants, bacteria, and fungi are used as stabilizers and reducing agents to control the growth of silver particles. In biosynthesis of silver nanoparticles, the biomass or biological system used normally contains functional groups with reducing capacity, and it interacts with preformed nanoclusters or nuclei of silver metal present in the system to form silver nanoparticles. Various resources used in this method have been reported [40] including peptides [41, 42], bacteria [43–46], and fungi [47–49]. This strategy has drawn great interests due to the ease of handling [40].

Photochemical preparation suggests another way of thinking silver nanoparticle growth. Generally, it uses light, i.e., UV light to transform colloidal solutions of spherical silver nanoparticles into stable larger nanoparticles with different shapes and sizes [50–52]. In photochemical method, solutions of colloid silver are prepared as the primary source of silver nanoparticles. This process usually involves photoreduction of silver salts, such as silver nitrate and silver perchlorate and the presence of polymer stabilizers including PVP, PMMA, and PMAA. The photochemical growth of silver nanoparticles can be controlled by choosing the light sources and the concentrations of polymer stabilizer [51, 53].

### Preparation of Nanofibers Containing Silver Nanoparticles

Ag (0)-polymer nanocomposites have been synthesized through different methods by introducing precursors of silver salts into polymer matrix followed by either chemical reduction or laser ablation and further processing to form silver-bonded polymer micelles or silver-embedded fibers.

Depending on the method of silver particle preparation, the synthesis of Ag (0)-polymer nanocomposites can be one step or two steps. In a one-step method, silver precursor and polymer, which serves as stabilizer for solver nanoparticles, share the same solvent. Selective solvents must be used in this method so that both the precursor and polymer can be dissolved. In addition, the solvents, normally are ethanol and methanol, should be able to reduce the silver precursors into silver nanoparticles. After a homogeneous solution system is obtained, it is then further subjected to electrospinning to produce silver nanoparticle-containing nanofibers. While in the two-step method, instead of being reduced by solvent, the transformation of the silver precursors into silver nanoparticles requires an additional step. In this method, the silver precursor, which is usually silver nitrate, is introduced to polymer solution. After a homogeneous dispersion is achieved, the solution is then subjected to laser ablation or chemical reduction. Plasma treatment has been reported to be an effective way to produce silver nanoparticle in nylon 6 solution [54]. Chemical reduction has also been employed to prepare silver nanoparticles in polypyrrole (PPy) solution [55]. At the second step, electrospinning is employed to make the silver particle suspended polymer solution into nanofiber composites. Although the one-step method is simpler and requires less treatments and processing than the two-step method, it is more selective to polymers and solvents. For the two-step method, because the silver particle reduction and nanofiber formation are performed in two separated steps, it does not need a solvent that is able to reduce silver precursors and there are more options for the solution systems. Theoretically, any polymer that is of interest can be used in the two-step method to load silver nanoparticles.

**Fig. 2:**
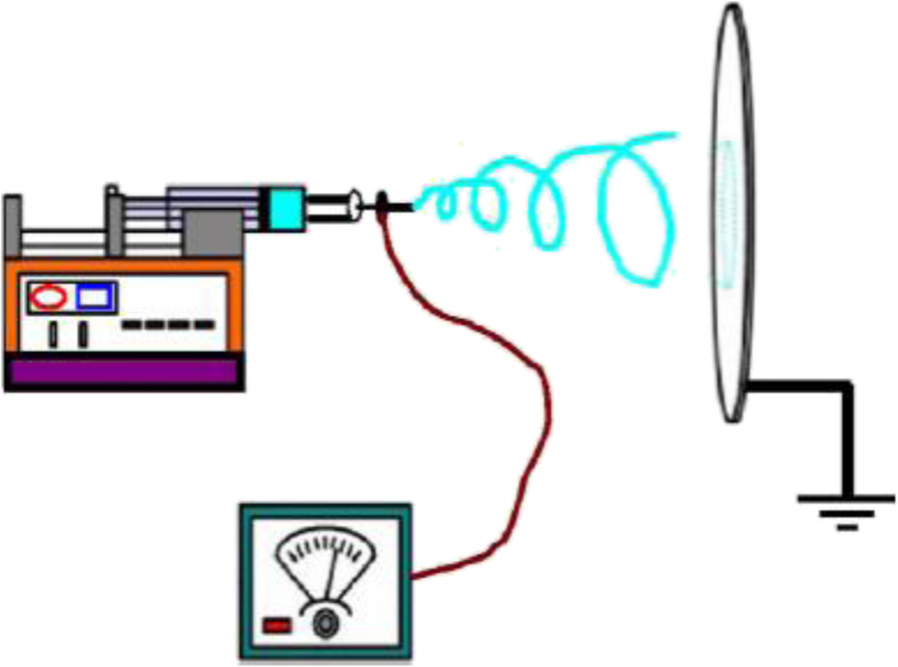
Schematic of electrospinning setup [53]

Electrospinning is a versatile and reliable technique to produce micro- or nanofibers. Electrospinning is a fiber forming process, where a high voltage is used to create an electrically charged jet of polymer solution or melt from the needle. When the voltage is high enough, the electrostatic forces overcome the surface tension of the polymer, and the jet is stretched and travels toward the collecting plate. The polymer solidifies during the traveling, often producing nanometer scale fibers. Nanofiber formation by electrospinning is affected by spinning parameters including solution properties and concentration, hydrostatic pressure in capillary tube, electric potential at the capillary tip, the tip-to-collector distance, and the chamber condition [56]. Figure 2 shows a regular electrospinning setup for nonwoven nanofibrous mats.

By electrospinning, the nanofibers embedded with silver nanoparticles can be prepared, and the morphology can be controlled by the electrospinning parameters. Another method involving in chemical reduction of silver particles requires only one step. In this method, precursors of silver salts are added into polymer solutions using reducing agent as the solvent, such as ethanol, methanol, and formic acid [57], while the polymer serves as the stabilizer during silver nanoparticle formation to prevent them from aggregating. This mixed silver precursor-polymer solution system is then objected to electrospinning to obtain silver-containing nanofibers.

**Fig. 3:**
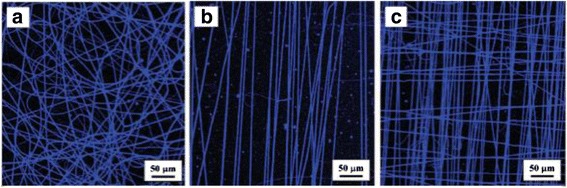
Confocal images silver-containing nanofibers with **a** nonwoven, **b** aligned, and **c** crossed structures [54]

By designing the geometries of the collecting target, nanofibers with various architectures can be obtained. It is reported that Ag/PVP nanofibers with nonwoven, aligned, and crossed patterns have been produced via coaxial electrospinning [58]. The resultant silver-containing nanofibers with different patterns are shown in Fig. 3 [58].

### Applications of Silver Nanoparticle-Containing Nanofibers

With merits of the antibacterial and fungicidal properties of silver nanoparticles and high surface-to-volume ratio of nanofibers, the application of the silver-containing nanofibers expands from biomedical applications to optical materials.

#### Antimicrobial Materials

**Fig. 4:**
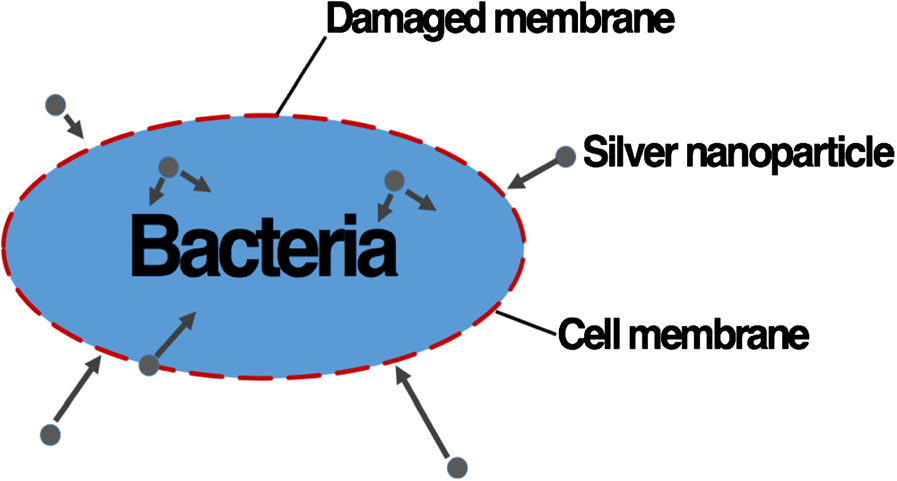
Illustration of the interaction between silver nanoparticles and bacterial cells

The mechanism of antibacterial function of silver nanoparticles is associated with the interaction between silver and the thiol group compound in bacterial fungal cells and fungus (Fig. 4). Although the exact mechanism remains unknown, it has been reported that structural changes are found in bacterial and fungal cells after the contact with silver nanoparticles. Comparing to regular silver particles, silver nanoparticles have favorable antibacterial and antifungal properties due to their extremely large surface area which allows better contact with microorganisms of bacteria and fungus. In addition, the silver nanoparticle gel not only attaches on cell membranes but also penetrates into the bacteria and fungus. After silver enters the cells, it binds to the cell wall and membrane and inhibits the respiration process [59]. In the case of *Escherichia coli*, the uptake of phosphate and releasing of mannitol, succinate, proline, and glutamine is inhibited by the presence of silver. Therefore, silver nanoparticles can be used as effective growth inhibitor in various microorganisms, and they are applicable to different antibacterial control system [60, 61].

**Fig. 5:**
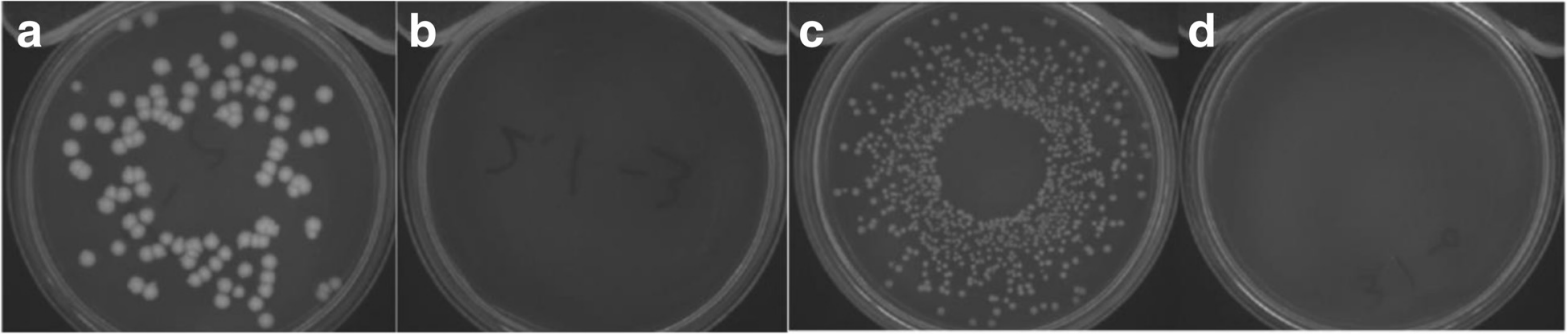
Antibacterial test plates of *Bacillus cereus* and *E. coli* **a**, **c** before and **b**, **d** after treatment with Ag/PAN nanofibers prepared from plasma-treated AgNO_3_/PAN solution. AgNO_3_ concentration 1.25 % [54, 57]

Studies have assessed the antibacterial performances of silver-containing nanofibers against both Gram-positive and Gram-negative microorganisms [54, 58]. In these studies, different polymers including nylon 6 and polyacrylonitrile (PAN) are used to prepare the silver-containing nanofibers. Although they are synthesized with one-step and two-step methods, respectively, both of these hybrid nanocomposites present promising antibacterial properties. Take nylon 6 as an example, the pure nylon 6 does not present any antibacterial activity, while after adopting silver nanoparticles in the polymer matrix, it shows a 99.9 % inhibition to *E. coli* when the silver precursor concentration is 0.5 wt.% and 99.9999 % inhibition when the concentration increases to 1.25 wt.% [57] (Fig. 5). These properties make the silver-containing nanofibers an excellent candidate for wound dressing and biotextile materials.

Researchers have also compared the antibacterial properties of silver-containing nanofibers with other commonly used polymers for wound healing without the presence of silver nanoparticles. The results show that the silver nanoparticle-coated polymer (PVA (polyvinyl alcohol)-Ag) possesses the best antibacterial performance and the highest healing efficiency as listed in Table 1 [62]. As it can be seen from the figure, PVA containing Ag nanoparticles exhibits the best healing power. The depth and area of wounds become the smallest among all the wounds treated with other nine membranes, including cotton gauze, PVA-crosslinked (crosslinked PVA), PVA-p-coated (wool protein-coated PVA), PVA-p-cospin (wool protein/PVA coelectrospun nanofibers), PCL (polyƐ-caprolactone), PCL-p-coated (wool protein-coated), PAN, PAN-PEU (polyurathane), and control (without using any wound dressings) after 16 days. This indicates that with the strongest antibacterial ability, the wounding area treated with PVA membranes containing silver nanoparticles recovers the best and fastest.

**Table 1:** Antibacterial effect assessment for different nanofiber membranes [82]

Sample groups	Bacteria growth	Antibacterial activity
Cotton gauze	Moderate growth	Insufficient effect
PVA-crosslinked	No growth	Good effect
PVA-Ag	No growth	Very good effect
PVA-p-cospin	Slight growth	Limit of efficacy
PAN	No growth	Good effect
PAN-PEU	No growth	Good effect
PVdF-HFP	Heavy growth	Insufficient effect

#### Photovoltaic Devices

The resonance effect of silver nanoparticles has been investigated and discussed by a large number of research. When the silver nanoparticles are paired with a dielectric material, the surface plasmons are excited with a combine character of electromagnetic wave and surface charge [63]. As strong light scattering elements, silver nanoparticles scatter lights and the light is then collected and trapped between the silver nanoparticles and dielectric thin films [11, 64]. Silver nanoparticle-containing nanofibers provide the environments of metallic nanoparticles and dielectric material (polymer), which has great potential to excite surface plasmons while compact in size, making it a perfect candidate for being used in organic solar cells to improve light absorption and trapping. It has been a great challenge to use thin polymer films to enhance the efficiency of light trapping and environment coupling for organic photovoltaic (OPV) devices. The silver-containing nanofibers make it possible to solve this long remained problem. Benefitted from the surface plasmon resonance effect of silver nanoparticles, and the mechanical flexibility and extremely high surface area of nanofibers, the energy harvesting is greatly enhanced. By employing the hybrid material of silver nanoparticle and PVP nanofibers as a thin layer in organic solar cells, research has suggested that an increase of 18.9 % in power conversion efficiency (PCE) has been reached. In this study, the nanofibers with three different patterns are prepared by electrospinning. They are nonwoven, aligned, and crossed, respectively. With confined geometries, charge drift velocity is improved, which further leads into smaller electrical resistance. In addition, the silver/PVP excites plasmon resonance with the nearby photoactive layer and leads to enhanced radiative energy transfers (Table 2).

**Table 2:** OPV characteristics with or without the Ag/PVP composite nanofibers [11]

Device	PCE (%)	R_s_ (Ω cm^2^)
Reference	3.53 ± 0.03	14.77
N-AgF-3	3.77 ± 0.09	13.84
A-AgF-3	3.97 ± 0.03	13.45
C-AgF-3	4.19 ± 0.03	12.29

Reference is OPV device without silver-containing nanofibers. The abbreviations of N-, A-, and C- are the ES nanofibers with the architectures of nonwoven, aligned, and crossed patterns

#### Catalyst for Hydrolysis/Electrolysis of Polymer Matrix

Another application to include silver nanoparticles in polymer nanofibers is to speed up the hydrolysis of the bulk material. Silver nanoparticles exhibit strong catalytic properties for hydrolysis and electrolysis of organic materials when being fabricated into silver nanoparticle/polymer composites [65, 66]. In addition, the catalytic activity can be tailored by controlling the size of silver particles and polymer matrix. Generally, the smaller particle size presents higher catalytic activity [66] with larger number of reaction sites, kink sites per surface area [67]. This size dependence becomes even more significant when the particle size shrinks to nanoscale. Studies have synthesized silver nanoparticles using a chemical reduction method, followed by embedding them into the polyvinyl acetate (PVAc) polymer matrix [68], to prepare silver nanoparticle PVAc nanocomposite. By introducing silver nanoparticles into polymer matrix, the hydrolysis of this hybrid material can be accelerated. The silver nanoparticles demonstrate a strong connection with the PVAc polymer chain, meaning that they can be remained in the matrix and continuously act as catalyst even when the backbone of polymer chains is broken down during hydrolysis. With the presence of silver nanoparticles, the hydrophobic PVAc polymer become hydrophilic and is subsequently dissolved in selective solvents, resulting in accelerated hydrolysis.

#### Water Filtration and Treatment

Among different kinds of nanosized antibacterial materials, i.e., ZnO, MgO, and TiO_2_, the silver nanoparticles have reported to be the most effective antimicrobial agent [69–77]. When paring with hydrogen-bonded multilayers assembled on magnetic microspheres, it can be delivered and localized in a specific region without contaminating the surrounding by using magnetic fields [78]. A composite of bifunctional Fe_3_O_4_ at silver nanoparticles with both superparamagnetic and antibacterial properties has been prepared and proved to have excellent antibacterial ability against *E. coli*, *Staphylococcus epidermis*, and *Bacillus subtilis* [79]. In addition to the benefits of supermagnetism, which allows the material to be easily removed from water, mesoporous polymer nanofiber membranes can be designed with specific pore sizes and desired filtration properties to enhance the water treatment efficiency and recyclability. The nanocomposites of supermagnetics/silver nanoparticle/polymer nanofiber can be a promising water disinfectant [79, 80].

#### Antimicrobial Nanopaints

Silver nanoparticles in silver nanoparticle/polymer nanocomposites are highly stable due to the polymer matrix. They can be sustained at up to 200 °C without significant oxidation and aggregation, which enables the production of silver nanoparticle embedded homogeneous paints. In the application of silver nanoparticle/polymer nanopaint, the silver nanoparticles are synthesized in polymer solution with the one-step method, followed by a drying process. The resultant silver nanoparticle-embedded drying oil is an excellent coating material and can be applied on various surfaces including wood, glass, and polystyrene [81]. The surface coated with the nanopaints exhibits outstanding antibacterial properties by killing both Gram-positive human pathogen and Gram-negative bacteria [81].

## Conclusions

Silver nanoparticles are widely used in biomedical materials because of their antibacterial property. Their applications also extend to optical and photovoltaic materials due to the light scattering effect on the metallic spherical particles and the potential to convert light into surface plasmon. Silver nanoparticles can be synthesized by chemical and physical methods with the presence of reducing agents, or laser ablation, respectively. The preparation of silver nanoparticles can be processed in selective polymers, i.e., PVP, PAA, and PVAc, and these polymers can be further prepared into nanofibers. Polymer nanofibers possess excellent mechanical properties and especially high surface-to-volume ratio and microporous structure, leading to larger number of reaction sites with chemicals and high permeability comparing to traditional fibers and membranes. Therefore, with the combined advantages of silver nanoparticles and polymer nanofibers, the hybrid material—silver nanoparticle-containing nanofibers and silver-containing polymer nanocomposites, has drawn tremendous interests for applications in biomaterials, catalysis, and photovoltaic materials. Electrospinning technique employs high voltage to prepare polymer solution into nanofibers. Preparation of polymer nanofibers containing silver nanoparticle becomes feasible via electrospinning. It can be one step or two steps depending on the process of reducing silver particles. In this work, the methods employed to prepare the hybrid material—silver nanoparticle-containing nanofibers and their applications, are presented and reviewed.

## Abbreviations

OPV: organic photovoltaic
PAA: polyacrylic acid
PAN: polyacrylonitrile
PCE: power conversion efficiency
PCL: polyƐ-caprolactone
PEG: polyethylene glycol
PEU: polyurathane
PMAA: polymethylacrylic acid
PMMA: polymethylmethacrylate
PPy: polypyrrole
PVA: polyvinyl alcohol
PVAc: polyvinyl acetate
PVP: polyvinylpropelene
